# Exploring the Nitrogen Ingestion of Aphids — A New Method Using Electrical Penetration Graph and ^15^N Labelling

**DOI:** 10.1371/journal.pone.0083085

**Published:** 2013-12-23

**Authors:** Franziska Kuhlmann, Sebastian E. W. Opitz, Erich Inselsbacher, Ulrika Ganeteg, Torgny Näsholm, Velemir Ninkovic

**Affiliations:** 1 Department of Ecology, Swedish University of Agricultural Sciences, Uppsala, Sweden; 2 Department of Forest Ecology and Management, Swedish University of Agricultural Sciences, Umeå, Sweden; 3 Department of Forest Genetics and Plant Physiology, Umeå Plant Science Centre, Swedish University of Agricultural Sciences, Umeå, Sweden; University College Dublin, Ireland

## Abstract

Studying plant-aphid interactions is challenging as aphid feeding is a complex process hidden in the plant tissue. Here we propose a combination of two well established methods to study nutrient acquisition by aphids focusing on the uptake of isotopically labelled nitrogen (^15^N). We combined the Electrical Penetration Graph (EPG) technique that allows detailed recording of aphid feeding behaviour and stable isotope ratio mass spectrometry (IRMS) to precisely measure the uptake of nitrogen. Bird cherry-oat aphids *Rhopalosiphum padi* L. (Hemiptera, Aphididae) fed for 24 h on barley plants (*Hordeum vulgare* L., cultivar Lina, Poaceae) that were cultivated with a ^15^N enriched nutrient solution. The time aphids fed in the phloem was strongly positive correlated with their ^15^N uptake. All other single behavioural phases were not correlated with ^15^N enrichment in the aphids, which corroborates their classification as non-feeding EPG phases. In addition, phloem-feeding and ^15^N enrichment of aphids was divided into two groups. One group spent only short time in the phloem phase and was unsuccessful in nitrogen acquisition, while the other group displayed longer phloem-feeding phases and was successful in nitrogen acquisition. This suggests that several factors such as the right feeding site, time span of feeding and individual conditions play a role for the aphids to acquire nutrients successfully. The power of this combination of methods for studying plant-aphid interactions is discussed.

## Introduction

Aphids are significant agricultural pests and have a strong economic impact due to their phloem-feeding behaviour [1], [2]. Aphids feed on phloem sap, entering the sieve tube to obtain carbohydrates, amino acids, minerals, vitamins and macromolecules [3]–[5]. Aphids' ability to rapidly reproduce causes a significant deprivation of plant nutrients [1], [2] and enhances virus transmission [6], [7]. To prevent aphid feeding, plants have developed a wide variety of defence mechanisms, e. g. morphological defence strategies such as trichomes and waxes, or chemical defences such as secondary plant metabolites and digestion inhibitors [8], [9]. Plant defence metabolites that are mobile in the phloem sap include phytohormones, polyamines [3], [10], species-specific secondary metabolites [11], [12] and defence proteins [5], [10], [13], [14]. Amino acids are the main nitrogen source for aphid growth and development, despite their low concentration in the phloem sap [15]. However, aphids can increase their nitrogen supply by forming symbiotic relationships with bacteria [15], [16] or by redirecting the plant's nitrogen allocation [17].

To relate aphid feeding to nutrient uptake, it is important to know whether an aphid is feeding or not. The Electrical Penetration Graph (EPG) technique is a well-established method to study aphid feeding behaviour in detail [9], [18], [19]. EPG was developed to overcome the limitations in studying aphid feeding without disturbing the aphid. For EPG experiments aphids and plants need to be connected to form a closed circuit during aphid feeding. For this connection, a golden wire is glued to the aphid and a copper electrode is attached to the soil near the roots of a potted plant [19]. When the aphid stylet penetrates the plant and starts feeding, a closed circuit is created. In this feeding process, different phases can be distinguished that show characteristic EPG waveforms [20], which are measured via a signal amplifier and recorder [19]. The movements of the stylet in the leaf tissue generate characteristic waveforms varying in amplitude, frequency and voltage [21]. Initial plant contact is characterised by stylet movements outside the phloem (labelled either with the letter C or F) summarised as stylet pathway phase. Stylet penetration into the phloem is defined as the phloem phase (phases E1 and E2) and stylet insertion into the xylem is called the xylem phase (phase G) [19]. Aphid feeding starts with intercellular probing (phase C) and cell puncture (pd, not listed separately) and is normally followed by the phloem phase E1, where saliva is injected into the sieve tube. Only after successful establishment in the sieve tube has the aphid entered the phloem-feeding phase (E2), in which it is able to feed continuously for hours or days [22]. Despite sieve tube puncture, the flow of phloem is maintained by continuously injecting watery saliva into the sieve tube [19], [21]. Salivation circumvents plant defence, which would plug the tube and induce callose sealing of sieve tubes, trigged by an increased calcium influx due to stylet penetration [21], [23]. Besides phloem-feeding, active drinking of xylem sap (phase G) can be observed, which maintains the water balance of the aphid [19], [24]–[27].

To investigate nitrogen uptake by arthropods in general and aphids in particular, tracer studies with artificial enriched plants or prey are widely used [28]–[31]. The stable ^14^N isotope has a natural abundance of 99.6337%, whereas the stable ^15^N isotope is rare with an abundance of 0.3663% [32]. Due to the low natural abundance of ^15^N, tracers enriched in ^15^N are widely used as stable, highly sensitive, non-radioactive markers in ecological and physiological studies [32], [33]. Nienstedt et al. were the first to analyse the ^15^N content of a single aphid, which had been feeding for two days on a plant fertilized with an ^15^N enriched nutrient solution. In this context it was also shown that the ^15^N label of the aphids increased with the grade of ^15^N enrichment of the host plant [28].

In this study we aimed to evaluate the combination of EPG and ^15^N labelling to estimate the amounts of N acquired by aphids feeding on barley plants, and to relate the duration and frequency of aphid-EPG-feeding phases to these amounts. We measured the ^15^N enrichment in single aphids after feeding on ^15^N labelled barley plants and correlated nitrogen enrichment with the time of the recorded EPG feeding phases within a time span up to 24 hours.

## Materials and Methods

### Plant growing and aphid rearing

Barley plants (*Hordeum vulgare* L., cultivar Lina) were cultivated under controlled conditions and moved regularly to prevent site effects and to give comparable plant growth. Barley seeds were pre-germinated in the dark and then transferred into pots (8×8 cm) filled with sterilized pumice stone (Bara Mineraler, Sweden) and grown for 13 days in a climate chamber (21/17°C day/night; 16/8 h light/dark; 200 µmol m^−2^ s^−1^; 60% humidity). Plants were fertilized every day either with a 6 mM NH_4_NO_3_ solution (Merck, unlabelled control plants) or a 6 mM ^15^NH_4_^15^NO_3_ solution containing 20% of labelled ^15^NH_4_^15^NO_3_ (^15^NH_4_^15^NO_3_, 98 atom%, Isotec, Miamisburg, USA, ^15^N labelled test plants) added to an essential micro- and macronutrients containing nutrient solution (pH = 5.8) according to Murashige and Skoog (MS medium) [34]. The applied volumes were adapted to plant age and were continuously increased starting with 2.5 ml three days after sowing to 20 ml at the end of the experimental period.

Bird cherry-oat aphids *Rhopalosiphum padi* L. (Hemiptera, Aphididae) were reared in cages on barley plants (cultivar Lina) grown on soil without fertiliser in a greenhouse (18–22°C; 16/8 h light/dark). Only wingless, adult aphids were taken for experimental analysis of ^15^N uptake.

To ensure equal light and rearing conditions, test and control barley plants were grown next to each other in the climate chamber, while the control aphids were reared in the greenhouse on barley plants potted in soil. Therefore control plants and control aphids represent a zero value in comparison with the ^15^N labelled test plants and test aphids.

### EPG (Electrical Penetration Graph) recording and EPG data analysis

At an age of 13 days, barley test plants were placed inside the Faraday cage of an EPG recording system that allowed simultaneous recording of EPG waveforms of 8 aphids (for details of the EPG recording system setup and software information see www.epgsystems.eu). The second leaf was fastened with PTFE strips and sticky tape to a flat surface to prevent leaf movement one hour before starting to record aphid feeding behaviour. The lower leaf side of ^15^NH_4_^15^NO_3_-fertilised plants was offered to the aphids and used for EPG recording. A gold wire connected to an electrode was fixed to the aphid with silver glue (recipe [35]). The recording of EPG waveforms started at noon and continued for 18 to 24 hours in the long time experiment (N = 22). In a shorter experiment the aphids (N = 8) were removed after 4 to 6 hours. For detailed information on the EPG data see Table S1.

Aphid feeding behaviour was analysed by the Stylet^+^ software provided by F.W. Tjallingii [35] for every aphid. Aphid feeding phases were defined after established definitions [35], E1 = phloem, salivation (we call it pre-phloem); E2 = phloem, ingestion; G = xylem, ingestion; C = stylet pathway activity, intercellular; F = derailed stylet mechanics; Np = non-probing. We additionally defined the variable feeding interruptions (FI) as the total number of stylet recordings in the non-feeding phases E1, G, C, F and Np in order to quantify feeding difficulties, especially the difficulty of aphids to establish themselves in the phloem feeding phase E2.

### Sample processing for stable nitrogen isotope analysis

Immediately after stopping EPG recording on ^15^N labelled barley plants, aphids were washed three times with distilled water to dissolve the silver glue which was removed with a paint brush. The cleaned aphids were individually sampled in a micro tube. Unlabelled control aphids were taken directly from their rearing plants and sampled in micro tubes (10 aphids pooled per experimental recording day). Total above ground plant biomass and the fresh weight of the second leaf of unlabelled and ^15^N labelled plants were determined, the second leaf was harvested and the plant and aphid samples were immediately frozen in liquid nitrogen and stored at −80°C until analysis. Prior to isotope analysis, the plant samples were freeze dried, weighed and homogenised with a mixing mill (Retsch MM 400, Germany). Subsequently, unlabelled control and ^15^N labelled aphids as well as aliquots of dried plant material (3–5 mg) were weighed in tin capsules (Säntis Analytical AG, Switzerland) for stable isotope analysis. Total N and ^15^N abundance were then measured by isotope ratio mass spectrometry (IRMS) using an elemental analyser (Flash EA 2000, Thermo Scientific, Bremen, Germany) connected in continuous flow-mode to a gas isotope ratio mass spectrometer (DELTA V Advantage, Thermo Scientific, Bremen, Germany). The abundance of ^15^N was calculated as δ^15^N [‰ vs. at-air] = (R_sample_/R_standard_−1)×1000, where R is the ratio of ^15^N/^14^N. The standard deviation of repeated measurements of a laboratory standard was 0.10‰ for δ^15^N.

The uptake of ^15^N by aphids feeding on ^15^N enriched plants was calculated as µg excess ^15^N uptake (based on atom percent excess values), after subtracting the natural ^15^N abundance of control aphids feeding on plants grown in soil, which was 0.369% [±0.001 SD, N = 6] ^15^N. The measured ^15^N atom % excess values in aphids were divided by 0.17145 to calculate the total nitrogen uptake based on the ^15^N values (atom%) measured in test plants (Table 1). The ^15^N content of offspring was not analysed since new born nymphs started eating immediately on their host plant. Therefore the influence of ^15^N loss of adults for incorporation into the offspring cannot be assessed.

**Table 1:** Nitrogen content in control/test barley plants and *R. padi* aphids (Mean ± standard deviation).

	plant		aphid	
	Control (N = 7)	Test (N = 29)	Control (N = 6)	Test (N = 30)
	mean ± SD	mean ± SD	mean ± SD	mean ± SD
**^15^N content [atom %]**	0.376±0.006	17.145±0.742	0.369±0.001	2.005±1.782
**^15^N abundance [δ^15^N ‰]**	27.0±15.3	55308±2902	6.8±2.3	4656±5142
**^15^N excess [µg]**	-	384.963±87.983	-	0.082±0.103
**total N [µg]**	2189±436	2288±475	4.2±1.6	4.7±2
**total N [mg gDW^−1^]**	68±8	77±2	90±14	80±16
**total N uptake [%]**	-	83.8±3.7	-	9.6±10.4

Different measures of nitrogen are presented as percentage of ^15^N [atom%]; abundance of ^15^N [δ^15^N ‰]; excess of ^15^N [µg] per plant or aphid; amount of total N [µg] per plant or aphid as well as concentration of total N [mg gDW^−1^] and uptake of total N [%] (% of total plant nitrogen coming from fertiliser or total aphid nitrogen coming from plant). Calculated values subtracted by the natural background label are ^15^N excess [µg] and total N uptake [%], therefore no values for control plants and aphids are given and rows are denoted by -.

### Statistical analyses

Statistical data analyses were performed with R version 3.0.0 (2013-04-03). For graphical visualization of the data SigmaPlot 11.0 (Systat Software) was used. Pearson product moment correlation coefficients were calculated and are given as correlation matrix. The response variable ‘µg excess ^15^N uptake’ was square root transformed to meet the assumptions of a linear regression model. Stepwise factor deletion was performed to obtain the minimal adequate model. For visualisation the square root transformed data was back-transformed and the regression lines were fitted to the original data.

## Results

Most of the aphids fed for 18 to 24 h (N = 22), whereas few aphids (N = 8) were removed already after a shorter time (4 to 6 h) to investigate the ^15^N uptake early after settlement on the plant.

Test barley plants were enriched on average by 17.145±0.742 atom % ^15^N after being cultivated on a ^15^N enriched nutrient solution, while in control plants this atom % value (0.376±0.006) was only slightly higher than the natural abundance of ^15^N (Table 1). The ^15^N content of test aphids was on average 2.005±1.782 atom % compared to 0.369±0.001 atom % of the control aphids. The test plants assimilated on average 384 µg excess ^15^N, from which it was calculated that 84% of their total nitrogen content was derived from the fertiliser solution. The test aphids ingested on average 0.082 µg excess ^15^N (and a maximum of 0.380 µg excess ^15^N), from which it was calculated that around 10% (and a maximum of 32%) of the total nitrogen content in the aphids was derived from the plant. The total amount of nitrogen per plant was on average 2.29 mg for the test plants (control plants: 2.19 mg) compared to 4.7 µg for test aphids (control aphids: 4.2 µg), which corresponds to 77 mg gDW^−1^ for test plants (control plants: 68 mg gDW^−1^) and 80 mg gDW^−1^ for test aphids (control aphids 90 mg gDW^−1^) (Table 1).

During the course of the experiment, 73% of the aphids produced offspring ranging from 1 to 5 nymphs, which on average had a mass of 5.6±2 µg dry weight per nymph. A significant correlation was found between the number of offspring and the ^15^N uptake of the adult aphids (Table 2). However, the number of offspring included as variable in the regression model (described below) had no significant explanatory function (Table 3).

**Table 2:** Pearson correlation coefficient matrix.

	excess ^15^N	E2	E1	G	C	F	FI	offspring	non-feeding
**excess ^15^N**	1.00	**-**	**-**	**-**	**-**	**-**	**-**	**-**	
**E2**	**0.86**	1.00	**-**	**-**	**-**	**-**	**-**	**-**	
**E1**	−0.24	−0.07	1.00	**-**	**-**	**-**	**-**	**-**	
**G**	−0.31	−0.29	0.33	1.00	**-**	**-**	**-**	**-**	
**C**	−0.31	−0.19	**0.52**	**0.59**	1.00	**-**	**-**	**-**	
**F**	−0.20	−0.22	−0.11	**0.40**	0.20	1.00	**-**	**-**	
**FI**	−0.31	−0.19	**0.46**	**0.55**	**0.97**	0.24	1.00	**-**	
**offspring**	**0.51**	**0.53**	0.28	0.22	**0.39**	−0.25	0.31	1.00	
**non-feeding**	**−0.36**	−0.26	**0.58**	**0.83**	**0.93**	0.35	**0.89**	0.34	1.00

Significant correlation coefficients (p≤0.05) are written in bold numbers. Abbreviations: E2 = phloem feeding, E1 = pre-phloem, G = xylem feeding, C = pathway phase, F = derailed stylet mechanics, FI = feeding interruptions, non-feeding = sum of E1, G, C and F phase.

**Table 3:** Steps of model simplification to find the minimal adequate model.

	linear additive model predictors								model summary			
sqrt (µg excess ^15^N)	intercept	E2	E1	FI	offspring	G	F	C	F-statistic	DF	r^2^	p
	0.0953	0.0178	−0.0227	−0.0002	0.0240	−0.0083	0.0300	−0.0034	23	7;22	0.84	<0.001
	0.0943	0.0180	−0.0237	−0.0004	0.0228	−0.0085	0.0298	-	28	6;23	0.85	<0.001
	0.1012	0.0184	−0.0278	−0.0003	0.0184	−0.0055	-	-	34	5;24	0.85	<0.001
	0.0960	0.0191	−0.0285	−0.0004	0.0159	-	-	-	43	4;25	0.85	<0.001
	0.0945	0.0212	−0.0239	−0.0003	-	-	-	-	54	3;26	0.85	<0.001
	0.0819	0.0216	−0.0339	-	-	-	-	-	78	2;26	0.84	<0.001

E2 = phloem feeding, E1 = pre-phloem, FI = feeding interruptions, G = xylem feeding, F = derailed stylet mechanics, C = pathway phase.

### ^15^N uptake correlates with the phloem-feeding duration of aphids

**Figure 1:**
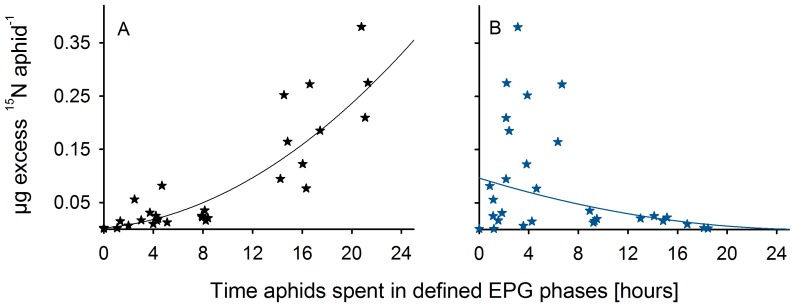
Stable ^15^N enrichment of aphids correlates with phloem-feeding duration. The calculated values of the µg excess ^15^N uptake of *R. padi* aphids (N = 30) are plotted against the time that aphids spent in defined EPG phases on barley plants. Either the phloem-feeding phase E2 (A) or the sum of defined non-feeding phases are plotted (B). The non-feeding time includes the time aphids spent cell puncturing (C), had derailed stylet mechanics (F), were xylem drinking (G) and saliva injecting (E1). Pearson's product moment correlation between ^15^N uptake and phloem-feeding A, t = 8.95, cor = 0.86, p<0.001 and ^15^N uptake and non-feeding B, t = −2.03, cor = −0.36, p = 0.05 (Table 2). The feeding experiment was conducted for up to 24 hours and the feeding behaviour was recorded with EPG. The µg excess ^15^N uptake of control aphids was zero.

The nitrogen uptake of aphids was positively correlated with the duration of phloem-feeding (Fig. 1 A, Table 2), whereas for other single EPG phases including E1 no significant correlation with ^15^N excess uptake was found. Summing up the time aphids spent in non-feeding phases (E1, G, C and F), a significant negative correlation between these non-feeding phases and ^15^N uptake was detected (Fig. 1 B and Table 2).

The positive correlation between ^15^N uptake per aphid (from now referred to [µg excess ^15^N aphid^−1^]) and the phloem-feeding duration (phase E2) was best explained by a linear regression based on the square root (sqrt) transformed [µg excess ^15^N aphid^−1^] data. Model simplification was used to identify the minimal adequate model. The maximal model contained the time aphids spent phloem-feeding (E2), in the pre-phloem (E1), in the xylem (G), in the pathway (C) phase and with derailed stylet mechanics (F) as well as offspring and feeding interruptions as model predictors. The minimal adequate model with the highest explanatory power was: *√y = 0.0819+0.0216×phloem feeding−0.0339×pre-phloem*; F _(2;26)_ = 78, r^2^ = 0.84, p<0.001. Hereby, the factors phloem-feeding and pre-phloem feeding E1 had a significant impact on the ^15^N uptake of aphids. Phloem-feeding correlates positively to the ^15^N uptake per aphid, whereas the time aphids spent in the pre-phloem phase was negatively correlated with the ^15^N uptake per aphid (Table 3).

### Low and high nitrogen uptake

Using descriptive statistics, the aphids can be subdivided in two groups according to their ^15^N acquisition. One aphid group took up only little ^15^N (N = 20) feeding for less than 9 h in the phloem phase, while the other aphid group (N = 10) was able to ingest high amounts of ^15^N feeding for at least 14 h (Fig. 1A).

**Figure 2:**
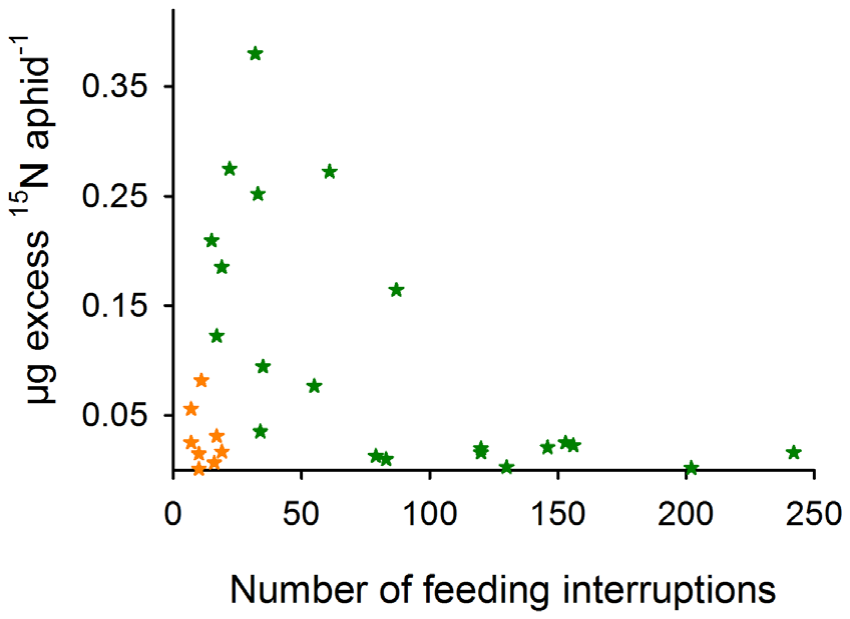
High ^15^N enrichment is characterised by few feeding interruptions. The µg excess ^15^N uptake of individual *R. padi* aphids is plotted against the number of feeding interruptions. Green stars (N = 21), experimental time up to 24 hours. Orange stars (N = 8), experimental time from 4 to 6 hours. A zero value of no feeding behaviour is excluded in the plot and the statistical analyses. Pearson's product moment correlation for 20 hours, t_(20)_ = −3.02, cor = −0.56, p = 0.007; for 4 to 6 hours, t_(6)_ = −0.73, cor = −0.28, p = 0.495.

Some aphids could not establish themselves in the phloem phase (E2) at all or fed in the phloem phase only for a short time (0.6±0.8 h aphid^−1^, N = 5), which resulted in a low ^15^N label (0.003±0.002 µg excess ^15^N aphid^−1^, N = 5) that was in the range of ^15^N levels of the control aphids (0±0.0003 µg excess ^15^N aphid^−1^, N = 6). Aphids that had been drinking mainly in the xylem for more than 5 h in combination with low phloem feeding activity (1.7±1.7 h aphid^−1^, N = 3) incorporated low ^15^N amounts (0.005±0.005 µg excess ^15^N aphid^−1^, N = 3). Numerous feeding interruptions (over 200 in 20 h were recorded) resulted also in low ^15^N values (0.009±0.007 µg excess ^15^N aphid^−1^, N = 2; Fig. 2), while the aphids were feeding in the phloem on average for 2.7±1.6 h.

On the other hand, aphids that fed most of the experimental time in the phloem showed a high ^15^N enrichment. Constant phloem-feeding (phase E2) for more than 15 h and only 33±16 interruptions per aphid resulted in a comparably high ^15^N uptake (0.278±0.056 µg excess ^15^N aphid^−1^, N = 5; Fig. 2). Those aphids took up around 25% of their total body nitrogen (26.5±6% total N uptake aphid^−1^) during on average 19 h phloem-feeding (18.9±2.8 h, N = 5) with a maximum value of 32% total nitrogen uptake in one aphid.

**Figure 3:**
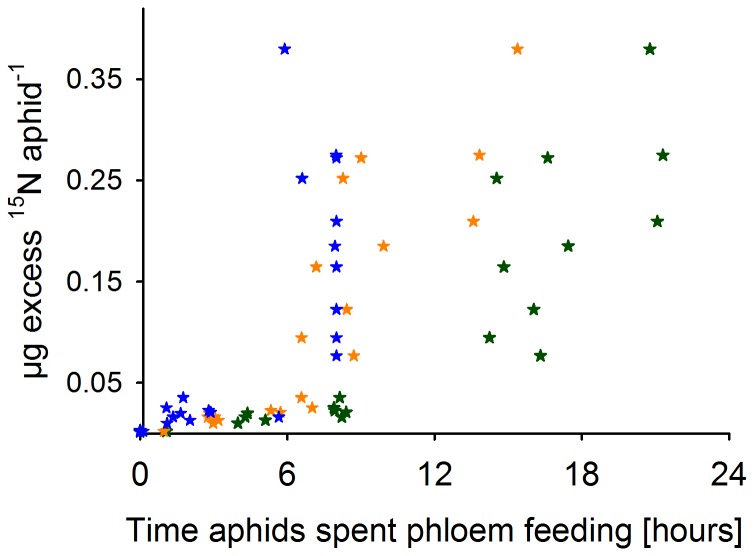
Long day and night phloem-feeding times are important for successful ^15^N uptake by aphids. The µg excess ^15^N uptake of individual *R. padi* aphids (N = 22) are plotted against the time aphids had been feeding in the phloem phase (E2) on barley plants either during night (blue stars), day (orange stars) or total (day and night; dark green stars). Day (16 hours) and night (8 hours) values of phloem-feeding are extracted out of the total aphid phloem-feeding duration (E2). The feeding experiment was conducted for up to 24 hours and the feeding behaviour was recorded with the EPG method. The µg excess ^15^N uptake of control aphids was zero.

In order to examine possible circadian influences on aphid feeding, the time aphids (N = 22) spent phloem-feeding (E2) at day and night was separately summed and plotted against the ^15^N uptake [µg excess ^15^N aphid^−1^] (see Fig. 3). The already mentioned grouping observed for successful and unsuccessful aphids is visible when only the feeding pattern during the night is considered (Fig. 3, blue stars). In contrast, the day feeding pattern showed no such grouping, but rather a continuous positive correlation between phloem feeding and µg ^15^N excess uptake (Fig. 3, orange stars). However, combining both day and night feeding periods, the grouping pattern is more pronounced (Fig. 3, green stars).

Most aphids (20 out of 22 aphids) started to feed already in the first light period (E2 phase longer than 10 min) and were also able to feed at least a little during the night. Only two aphids failed to reach the phloem during both day or night. Continuous phloem feeding was observed for ten aphids that were feeding over 14 h starting at day, and most of them (8 out of 10) fed throughout the night. Drinking behaviour in the xylem phase (G) was investigated, and 17 out of 22 aphids drank under the light period, while 11 out of those 17 aphids drank additionally at night. Only five aphids drank neither at day nor at night. The drinking periods were longer at day than at night, though the variation was high (0 to more than 7 h at day and 0 to 3 h at night time).

## Discussion

Aphids are major pests on plants; through feeding on phloem sap they cause a range of negative effects, not the least through removing nutrients, in particular nitrogen from the plant [36]–[38]. The complex feeding behaviour of aphids has been described in detail using the EPG technique through which different phases of aphid feeding can be specified [19], [20]. However, this technique provides only qualitative data on feeding behaviour, and the link between feeding behaviour and quantitative data on ingested amounts of nutrients has not been described. Our study aimed at filling this gap by combining the EPG technique with quantification of amounts of ingested N through the use of the stable isotope ^15^N. Our data (Fig. 1) provide clear evidence that the phloem-feeding phase (E2) actually corresponds to ingestion of nutrients. However, aphids spending less than 9 hours in the E2 phase displayed very low enrichment of ^15^N, suggesting the existence of a threshold in the length of time aphids need to spend feeding in the phloem. A necessity for the largest possible nitrogen uptake is an early successful establishment in the E2 phase and continuous feeding over a longer time period. The slope of the relationship between ^15^N enrichment versus time spent in phase E2 corresponds to the efficiency of feeding. The presented technique combines two powerful methods to study the feeding behaviour of individual aphids, avoiding radioactive labelling and any negative impact on the survival or fertility of both plants and aphids [28]. This method combination gives the opportunity to answer various questions concerning the plant perspective (e.g. plant nitrogen physiology), the aphid perspective (e.g. nitrogen uptake, metabolism of specific nitrogen containing compounds) or plant-aphid interactions (e.g. allocation of nutrients, discrimination of substances).

Test plants and test aphids were highly enriched with ^15^N compared to their respective controls. The very low variation within the test plants confirms the robustness of stable isotope ratio mass spectrometry, as plant homogeneity is important for studying nutrient uptake in aphids (Table 1). A high percentage (84%) of assimilated nitrogen was found to originate from the fertiliser solution, while the other part may come from the seedling. On the other hand, high variation in the ^15^N signature for the test aphids was dependent on how successfully individual aphids established themselves in the phloem of the test plants. The loss of nitrogen due to honeydew excretion [24], [39] and offspring production [28] may also contribute to this variation.

For the control samples, the small difference in ^15^N values between plants and aphids can be explained by isotope fractionation during the feeding process. Compared to their diet, fluid feeding insects can be depleted in ^15^N up to 10‰ [33]. Therefore small differences in the ^15^N signature were expected, especially because control aphids had not been feeding on control plants. However, droplet contamination of control plants with ^15^N enriched fertiliser cannot be ruled out.

Already after less than one day of phloem-feeding on barley plants fertilised with a 20 atom% ^15^N nutrient solution, high amounts of nitrogen were taken up with a maximum of 32%. In one aphid a ^15^N enrichment 15 times higher than the natural abundance in control aphids was measured. Nienstedt et al. [28] measured 25 to 90 times higher ^15^N acquisition in aphids after 7 or 11 days of feeding on plants fertilised with a 50 atom% nutrient solution. These values are not easily comparable as ^15^N uptake depends on the individual feeding behaviour of the aphids. In our experiment, the high ^15^N amounts in aphids suggest that shorter feeding times or lower fertiliser labelling is sufficient to measure phloem sap ingestion of aphids. The ^15^N label may be reduced to approximately 2 atom% ^15^NH_4_^15^NO_3_ in the nutrient solution and a resulting 10 times lower ^15^N signature would still allow robust analysis to study aphid nutrient uptake. However, when focussing on the identity of ingested nitrogen containing compounds, a high plant labelling might be more suitable to ensure maximum concentrations for mass-spectrometric identification of e.g. alkaloids [12]. According to the requirements in a given experiment, the desired ^15^N labelling is easily adjustable.

### ^15^N uptake correlates with phloem-feeding duration of aphids

According to our results, the major proportion of nitrogenous compounds is taken up by the aphids while feeding in the phloem phase E2 [3], [20]. All stylet activities other than phloem-feeding in phase E2 do not contribute to the nitrogen ingestion of aphids, the time aphids spent in the pre-phloem phase E1 is even negatively correlated. In compliance with the regression analyses, over 80% of the variance of aphid ^15^N uptake can be explained by the phloem-feeding phase (E2) (Fig. 1, Table 2).

As early as 1978, similar results for aphid feeding behaviour were published using radioactive ^32^P labelling [18]. Also here the EPG method was used to study aphid feeding behaviour in detail and *Brevicoryne brassicae* was allowed to feed on the ^32^P labelled artificial diet for 2 hours. At this time the feeding phases pre-phloem (E1), phloem (E2) and xylem (G) had not been distinguished from each other and were summarised in one feeding phase. The uptake of ^32^P in *B. brassicae* was clearly correlated to the feeding phase and only a much lower yet significant correlation was found with the time spent in the pathway phase. Our results are in line with Tjallingii even though we found no correlation between the ^15^N uptake and the pathway phase C (Table 2) [18]. As the cited study was conducted on artificial diet and not on a living plant a comparison is difficult. Since then, the EPG method has improved greatly and aphid feeding behaviour has been unravelled in detail, facilitating the understanding of the relationship between phloem-feeding and nitrogen uptake in this study.

It is widely accepted that phloem transport of nitrogen is in organic forms, mainly as amino acids [40]. Regardless of the source, nitrogen can be incorporated in amino acids, which are subsequently transported through the phloem or xylem to other tissues [41]–[43]. Besides the known phloem transport, labeled amino acids can also be transported in the xylem of barley plants [44]. Therefore, it is possible that xylem drinking and xylem-amino acids respectively may increase the ^15^N label in the aphids. Winter et al. [45] reported a total amino acid concentration of over 200 mM in the phloem sap of barley, whereas Seel et al. [44] quantified only concentrations of about 5 mM in the xylem sap. Our data according to the model simplification do not support a nitrogen uptake of the aphids via the xylem (Table 2), which may be the result of the very low amino acid concentrations in xylem sap compared with those of phloem sap. The aphids that spent many hours xylem drinking had very low ^15^N values that were in the range of the control aphids.

The observed variation in ^15^N uptake may be explained by several other factors. The wired aphids had only a restricted choice of suitable feeding sites among differently sized phloem vessels, which might affect nitrogen uptake. Further, defence responses of the plant e.g. callose plugging of sieve tubes might affect ^15^N uptake by hindering the establishment of aphids in the phloem phase E2 [17], [21]. This might have caused the very low ^15^N enrichment of aphids that were unable to reach the phloem. During the experiment several adults produced offspring, which may also influence the nitrogen enrichment. Since new born aphids were not removed, and they usually start feeding immediately on their host plant, we did not analyse their ^15^N signature. ^15^N enrichment in adult aphids may was pronounced when offspring maturation began before the experiment on non-labelled plants, and therefore predominantly ^14^N was incorporated in the offspring. Alternatively, ^15^N enrichment was reduced when mainly recently ingested ^15^N was transferred to the offspring. Nienstedt et al. [28] observed a transfer of labelled ^15^N from adults to their offspring, although the extent was comparably low. Given the little information about the label of the offspring, it is difficult to discuss the influence of their number and weight on a putative nitrogen loss of the adults. However, we could find a significant positive correlation of offspring number and the adult ^15^N label, which indicates that high offspring productions is connected to a high nitrogen ingestion (Table 2).

### Low and high ^15^N enrichment

Although a strong correlation between phloem sap feeding and nitrogen acquisition was found, further information is difficult to extract. The correlation between nitrogen uptake and phloem-feeding (E2) suggests the division of aphids into two groups (Fig. 3). One group with low ^15^N enrichment showed a non-continuous phloem-feeding behaviour combined with a frequent change between stylet penetration activities outside and inside the phloem phase as well as xylem drinking. This group had feeding problems, and feeding interruptions can cause a break in ^15^N uptake that reduces the isotopic enrichment of aphids (Fig. 2). The group showing a high ^15^N uptake mostly fed throughout the night and also during long phases at day (Fig. 3). Presumably, the earlier and longer an aphid is able to feed continuously, the higher the ^15^N enrichment will be. This indicates that a specific threshold of phloem-feeding time is needed, after which phloem sap ingestion is facilitated. For successful phloem feeding, aphids need to circumvent plant defence responses, e.g. by salivation in the early phases of phloem establishment [21], [23].

Since most of the aphids were investigated over a day-night cycle, this circadian change might have caused some variation in aphid feeding behaviour. However, we cannot clearly identify a circadian pattern in the nitrogen uptake of the aphids. It is a matter of discussion whether circadian rhythm can influence food ingestion in aphids. In barley plants (*Hordeum vulgare* L.) amino acid concentrations had been higher in the dark period compared to the light period therefore aphids should ingest more nitrogen during the night [45]. Studies recording the honeydew production of aphids suggest a clear circadian rhythm in aphid feeding due to differences in carbohydrate [46], [47] and nitrogen concentrations [48] of the phloem sap. In other plant species, e.g. tansy (*Tanacetum vulgare* L.; Asteraceae) and castor bean (*Ricinus communis* L., Euphorbiaceae) no circadian rhythm was found in the sucrose:amino acid ratio of phloem sap [49], while in potato plants (*Solanum tuberosum* L., Solanaceae) the sucrose:amino acid ratio of phloem sap was lowest at dawn [48]. This suggests that focus should be put on plant-aphid interaction itself, to examine a possible direct impact on the circadian rhythm of plant nutrient allocation as well as aphid food uptake simultaneously. Our proposed method gives the opportunity to investigate a possible day-night influence, when aphids will be sampled separately at either day or night on ^15^N labelled plants.

### Conclusion

In conclusion, we show that the more phloem sap (E2 phase) is ingested, the higher the aphids are enriched in ^15^N due to the uptake of nitrogenous compounds. Through the combination of EPG behavioural recording and ^15^N isotope mass spectrometry analysis, the food ingestion of aphids can be monitored in detail. Nitrogen uptake can be related to a precise feeding history explaining food uptake. This method can give to deeper understanding of the individual roles of the on-going battle between the ‘defending’ plant and the ‘attacking’ aphid, especially when focussing on the plant's nutrient allocation at day and night. The influence of the plant's circadian clock on nutrient supply to the aphids cannot be clarified and further studies are needed.

## Supporting Information

Table S1 EPG data of all analysed aphids, see supporting information.
(XLSX)
